# Dietary Fiber as a Counterbalance to Age-Related Microglial Cell Dysfunction

**DOI:** 10.3389/fnut.2022.835824

**Published:** 2022-03-14

**Authors:** Mario Vailati-Riboni, Laurie Rund, Maria Elisa Caetano-Silva, Noah T. Hutchinson, Selena S. Wang, Katiria Soto-Díaz, Jeffrey A. Woods, Andrew J. Steelman, Rodney W. Johnson

**Affiliations:** ^1^Department of Animal Sciences, University of Illinois at Urbana-Champaign, Urbana, IL, United States; ^2^Division of Nutritional Sciences, University of Illinois at Urbana-Champaign, Urbana, IL, United States; ^3^Department of Kinesiology and Community Health, University of Illinois at Urbana-Champaign, Urbana, IL, United States

**Keywords:** aging, fiber, gut-brain axis, microglia, neuroinflammation, short-chain fatty acids

## Abstract

With increasing age, microglia shift toward a pro-inflammatory phenotype that may predispose individuals to neurodegenerative disease. Because fiber fermentation in the colon produces bioactive short-chain fatty acids (SCFAs; e.g., acetate, butyrate, and propionate) that signal through the gut-brain axis, increasing dietary fiber may prevent or reverse age-related dysregulation of microglia. Adult (3–4 months old) and aged (23–24 months old) male and female mice were given *ad libitum* access to a modified AIN-93M diet with 1% cellulose or the same diet with 2.5 or 5.0% inulin for 8 weeks. Several adult and aged male mice fed 0 or 5% inulin were randomly selected for whole brain single-cell RNA sequencing (scRNA-seq) and differential gene expression analysis to classify brain microglia according to gene expression profile; and identify additional genetic markers of aging as possible targets for dietary interventions. Microglia were isolated from remaining mice and expression of selected aging-, inflammatory-, and sensome-related genes was assessed by Fluidigm as was the *ex vivo* secretion of tumor necrosis factor-alpha (TNF-α). SCFAs were measured in samples collected from the cecum. Microglia from adult and aged mice segregated into distinct phenotypes according to their gene expression profile. In aged mice, a considerably greater proportion of the population of microglia was identified being “activated” and a considerably smaller proportion was identified being “quiescent.” These findings using whole brain scRNA-seq were largely corroborated using highly purified microglia and Fluidigm analysis to assess a selected panel of genes. Aged mice compared to adults had lower levels of SCFA’s in cecum. Dietary inulin increased SCFAs in cecum and mostly restored microglial cell gene expression and TNF-α secretion to that seen in adults. Sex differences were observed with females having lower levels of SCFAs in cecum and increased neuroinflammation. Overall, these data support the use of fiber supplementation as a strategy to counterbalance the age-related microglial dysregulation.

## Introduction

In the brain of young healthy adults, non-inflammatory microglia constantly survey the parenchyma and maintain homeostasis (1). If microglia encounter harmful endogenous ligands (danger associated molecular patterns) and microbes (pathogen associated molecular patterns), they react and display pro-inflammatory activity to remove the insult followed by anti-inflammatory activity to promote defense and healing (2, 3). However, aging predisposes brain microglia to a pro-inflammatory state that results in chronic neuroinflammation and heightened vigilance (3, 4). Most studies indicate aged microglia lose their neuroprotective functions, which is consistent with a report indicating aged microglia retain a prominent pro-inflammatory profile and are less sensitive to the anti-inflammatory effects of IL-4 (5). Chronic age-related neuroinflammation increases risk for mood disorders such as anxiety and depression (6). It also results in diminished cognitive abilities (7), and further predisposes individuals to neurodegenerative diseases like Alzheimer’s (8), and reduces the likelihood for independent living (9). Therefore, strategies to normalize microglia may delay age-associated neural pathologies and neurodegenerative disease.

While the explanation for why microglia become pro-inflammatory during aging is unclear, intestinal dysbiosis and altered viscerosensory signaling may be involved since intestinal microbiota have been shown to regulate microglial cell activity, neuroinflammation, and neurodegenerative disease (10–12). It is believed that gut microbiota impart some of their effects by fermenting dietary fiber in the colon to produce bioactive short-chain fatty acids (SCFAs) including acetate, butyrate and propionate. Among the SCFAs, butyrate has been extensively studied and, in addition to being an energy substrate for colonocytes (13) and maintaining intestinal homeostasis (14), it has been shown to have pro-cognitive effects, at least when given at pharmacological doses (15), and to inhibit microglia activation *in vitro* (14, 16). However, little is known about dietary fiber, SCFAs, and age-related neuroinflammation. Nevertheless, this is important because older men and women consume far below the recommended daily level of fiber (17), and display a reduced fermentation capacity such that, at similar levels of intake, their microbiota produces less SCFA compared to adults (18, 19).

In the current study, we sought to better understand how aging and dietary fiber affect microglia. First, we used whole brain single-cell RNA sequencing (scRNA-seq) and differential gene expression analysis to (1) classify brain microglia from adult and aged mice according to their gene expression profile; and (2) identify additional genetic markers of aging as possible targets for dietary interventions. Subsequently, we determined in young adult and aged male and female mice the effect of dietary inulin on brain microglia. Inulin, a prebiotic fermentable fiber increases endogenous production of SCFA, especially butyrate, by increasing butyrate-producing bacteria (20). The important results identified two large distinct microglial cell clusters classified as quiescent or activated with the latter much more pronounced in aged mice compared to adults, coupled with the lower production of SCFA by aged animals. Moreover, the results support the use of dietary fiber to increase SCFA concentrations and limit age-related neuroinflammation.

## Materials and Methods

### Animals, Dietary Treatments, and Tissue Collection

All animal care and handling procedures were approved by the University of Illinois Institutional Animal Care and Use Committee and were in accordance with federal guidelines. Male and female C57BL/6J mice were obtained at 1–2 months of age (in-house colony) and 21–22 months of age (National Institute on Aging, Bethesda, MD, United States). To bring gut microbiota into conformity, mice were kept in the same room under a reverse 12 h light:dark cycle and given *ad libitum* access to water and AIN-93M diet (Envigo, Indianapolis, IN, United States) for 8 weeks. During weekly cage changes, used bedding (e.g., litter and feces) from mice similar in age was collected, homogenized, mixed with clean bedding, and redistributed to each cage. Body weights (BW) were recorded weekly.

Following the acclimation period, adult (3–4 months old) and aged (23–24 months old) male and female mice were given *ad libitum* access to a modified AIN-93M diet with 1% cellulose or the same diet with 2.5%, or 5.0% inulin (Envigo, Indianapolis, IN, United States) for 8 weeks. Thus, the 12 treatment groups comprised the 2 × 3 × 2 factorial arrangement of age (adult and aged), diet (0, 2.5, or 5.0% inulin), and sex (male and female). Four hours after the onset of the dark phase mice were euthanized by CO_2_ asphyxiation and transcardially perfused with sterile ice-cold phosphate buffered saline (PBS). Brain tissue was collected and used immediately for scRNA-seq analysis (*Cohort 1*) or microglia isolation (*Cohort 2* and *3*). Contents were collected from the cecum, weighed, diluted 1:5 with a 6.25% metaphosphoric acid (e.g., 4 mL acid per 1 g gut contents), and stored at −20°C until SCFA analysis. Treatments comprised the 2 × 3 × 2 factorial arrangement of age (adult or aged), diet (0% = F0, 2.5% inulin, = F2.5, or 5% inulin = F5), and sex (female or male). The number of treatment replicates (*n*) for each variable studied is reported in the footnote of the respective figure or table.

**FIGURE 1:**
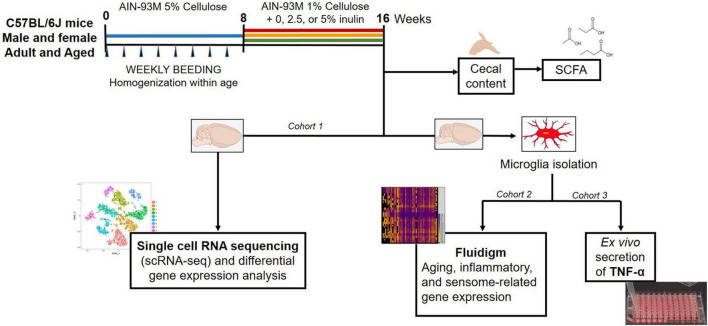
Schematic diagram of the experimental design.

Figure 1 shows a schematic diagram of the experimental design.

### Single-Cell RNA Sequencing and Analysis

#### Tissue Dissociation, Library Preparation, and Sequencing

Two adult and two aged male mice fed 0 or 5% inulin were randomly selected for scRNA analysis (*Cohort 1*). Whole brains were dissociated for 30 min at 37°C using the Adult Brain Dissociation kit (Miltenyi Biotec, Germany). After passing through a 70-μm cell strainer, debris and red blood cells were removed according to the kit protocol and the resulting pellet was sequentially depleted of myelin using the Myelin Removal Beads v2, and dead cells using the Dead Cell Removal Kit (both from Miltenyi Biotec, Germany). Final pellets were eluted in 100 μL of PBS with 4% bovine serum albumin (BSA). Single-cell 3′ cDNA libraries were prepared at the DNA Services laboratory of the Roy J. Carver Biotechnology Center at the University of Illinois at Urbana-Champaign. Acridine orange and propidium iodine staining on a Nexcelom K2 Cell Counter (Nexcelom Bioscience, Lawrence, MA, United States) indicated the four single-cell suspensions had an average cell viability of 75.5 ± 3.1% and contained 753 ± 121.3 cells/μL. Cells were converted into individually barcoded cDNA libraries with the Single-Cell 3′ Chromium kit version 3 from 10X Genomics (Pleasanton, CA, United States) following the manufacturer’s protocols. The target number of cells per library was 6,000. On average 14.5 ± 2.2 μL of the cell suspensions were used for the preparation of the libraries. The 10X Chromium instrument separated single cells into Gel Bead Emulsions (GEMs) to barcode the mRNA from each individual cell. Following ds-cDNA synthesis, individually barcoded libraries compatible with the Illumina chemistry were constructed. Each library was quantitated on Qubit and the average size was determined on an AATI Fragment Analyzer (Advanced Analytics, Ames, IA, United States). These four libraries were pooled evenly, and the final pool was diluted to 5 nM concentration and further quantitated by qPCR on a Bio-Rad CFX Connect Real-Time System (Bio-Rad Laboratories, Inc., CA, United States). This final library pool was sequenced on one lane of an Illumina NovaSeq 6000 S4 flowcell as paired-reads with 28 cycles for read 1, 8 cycles for the index read, and 150 cycles for read 2 (21). An average of 747,670,757 reads were obtained per sample.

#### Data Processing

Post processing of sequencing reads was conducted on a Biocluster2, the High Performance Computing resource of the Carl R. Woese Institute for Genomic Biology at the University of Illinois at Urbana-Champaign. Sequencing results were demultiplexed using the *mkfastq* function in Cell Ranger v3.1.0 and a filtered reference was created using the *mkgt* function starting from the *Mus musculus* GRCm38.98 ensembl build. The selected transcript biotypes for analysis were protein coding, antisense, long intergenic ncRNA, and Immunoglobulin and T cell receptor. Reads alignment, filtering, and barcode count was performed using the custom filtered reference *via* the *cellranger count* pipeline. Count matrices were then processed using R v3.6.0, and the Seurat v3.0 suite for single cell genomics (22, 23). After checking for overlapping barcodes, genes not detected in at least 10 cells were removed, and samples merged into one object. Cells were then filtered based on percentage of mitochondrial genes and total gene count, to detect dead cells or doublets, respectively. A threshold of three median absolute deviations was used for filtering. Filtered data were normalized using the *sctransform* method, and a principal component analysis was run using the *RunPCA* function. Clusters were identified using the first 40 principal components *via* the *FindNeighbors* and *FindClusters* function. Cluster cell type was identified combining classic biomarker analysis, and automatic predictions obtained by the SingleR package (24).

### Short-Chain Fatty Acid Analysis

Cecum samples (*Cohort 2*) were analyzed on an Agilent 7890 (Agilent Inc., Palo Alto, CA, United States) gas chromatograph, with an Agilent 5975 mass selective detector and Agilent 7683B autosampler. One microliter of sample was injected in a split mode (15:1), and analyzed on a 30 m HP-INNOWAX column with 0.25 mm inner diameter (I.D.) and 0.25 μm film thickness (Agilent, Palo Alto, CA, United States) with an injection temperature of 200°C, MSD transfer line of 200°C, and the ion source adjusted to 230°C. The helium carrier gas was set at a constant flow rate of 1 ml min^–1^. The temperature program was 2 min at 70°C, followed by an oven temperature ramp of 10°C min^–1^ to 190°C and 40°C to 240°C for a final 2 min. The mass spectrometer operated in positive electron impact mode (EI) at 69.9 eV ionization energy in m/z 30–300 scan range in combined scan and selected ion monitoring (SIM) modes. SIM targeted m/z 43, 45, 46, 60, 74. Target peaks were evaluated using Mass Hunter Quantitative Analysis B.08.00 (Agilent Inc., United States) software. Standard curves were generated for 0.1–50 mg L^–1^ range. At collection, an aliquot of each sample was weighed and used for dry matter (DM) analysis. Concentrations obtained from the chromatographer were then corrected for DM content and expressed as mmol g ^–1^.

### Microglia Isolation and Fluidigm Gene Expression Analysis

Brain microglia of mice from *Cohort 2* and *3* were isolated using a procedure adapted from Nikodemova and Watters (25). Brains were enzymatically digested using the Neural Tissue Dissociation Kit (P) (Miltenyi Biotec, Germany) for 22 min at 37°C. Further processing was performed at 4°C. Tissue debris was removed by passing the cell suspension through a 70-μm cell strainer. After myelin removal using 30% Percoll Plus (GE Healthcare, Princeton, NJ, United States), cells in PBS supplemented with 0.5% BSA and 2 mM ethylenediaminetetraacetic acid (EDTA) were incubated for 15 min with anti-Cd11b magnetic beads (Miltenyi Biotec, Germany). CD11b^+^ cells were extensively washed and separated in a magnetic field using MS columns (Miltenyi Biotec, Germany) before being directly placed in TRIzol reagent (Invitrogen, Carlsbad, CA, United States). Cells were then sonicated and stored at −80°C until further analysis.

Cell homogenates from *Cohort 2* were thawed on ice, and total RNA extracted using a commercially available kit (DirectZol, Zymo Research), following manufacturer’s protocols. RNA was then standardized to a final concentration of 35 ng/μL and converted to cDNA using the High-Capacity cDNA Reverse Transcription Kit (Thermo Fisher, Waltham, MA, United States), with the inclusion of Oligo dT18 (Integrated DNA Technologies, Coralville, IA, United States). Samples were then submitted to the University of Illinois at Urbana-Champaign Functional Genomics Unit of the W.M. Keck Center for Fluidigm analysis, using a 96 × 96 chip and two technical replicates for each combination of sample and assay. After an initial pre-amplification using a pool of all primers, data were acquired using the Fluidigm Real-Time PCR Analysis software 3.0.2 (Fluidigm, San Francisco, CA, United States). The run included a negative control and a standard curve obtained from a pool of all cDNA that was serially diluted 1:5. Data from Fluidigm runs were manually checked for reaction quality before analysis, and Ct values for each gene target were interpolated to a standard curve obtaining relative quantities. The relative standard curve method was chosen to control reaction efficiency and allow the implementation of a multifactorial model during statistical analysis. Relative mRNA quantities were then normalized to the geometric mean of the relative quantity of two housekeeping genes (*ACTB* and *GAPDH*), and log_2_ transformed prior to statistical analysis. Information on primers used are reported in Supplementary Table 1.

### Microglia Culture and Measurement of Tumor Necrosis Factor-Alpha and Interleukin 10 Proteins

Microglia from male adult and aged mice fed 0 or 2.5% inulin (*Cohort 3*) were isolated as described. Cells were resuspended in Dulbecco’s Modified Eagle Medium (DMEM) supplemented with 4 mM L-glutamine, 1 mM sodium pyruvate, 10% FBS, 1% Penicillin–Streptomycin (10,000 U/mL), at 5 × 10^5^ cells/mL and plated in a 96-well plate. Microglia were incubated at 37°C in a humidified incubator under 5% CO_2_ for 36 h. Supernatants were collected, centrifuged to remove debris, and stored at −80°C until assaying for tumor necrosis factor-alpha (TNF-α) and interleukin 10 (IL-10) using ELISA kits (Thermo Fisher, cat 88-7324-88 for TNFα; cat 88-7105-22 for IL-10) following the manufacturer’s instructions.

### Statistical Analysis

Differential expression of genes for the scRNA-seq analysis was analyzed using R v3.6.0 and the Seurat v3 package, and was limited for the purpose of this manuscript to the clusters identified as microglia (e.g., clusters 1, 5, and 14). The *FindMarkers* function within Seurat was used to conduct the statistical analysis, using the normalized data, without percentage presence and fold-change thresholds. The same function was used to find unique expression markers of the individual clusters to better identify microglia subpopulations. Genes were considered differentially expressed at FDR < 0.05, and FC > | 1.5|.

Gene Ontology (GO) analysis was done using the Panther Classification System^1^ using the list of differentially expressed genes as enrichment list, the list of all detected genes by the scRNA-seq analysis as background reference list, *M. musculus* as organism, and “GO Biological process complete” as functional classification. Fisher’s exact test was chosen for the statistical test, and a Bonferroni correction for multiple testing was applied. Subsequently, the list of statistically enriched processes was processed *via* REVIGO (26) for summarization and redundancy reduction. Processes were considered enriched with a corrected *P* < 0.05. For visualization purposes the enrichment score (−log_10_ *P*-value) was chosen.

Weekly BW, cecal SCFA corrected concentrations, and Fluidigm gene expression data were analyzed using SAS v9.4. All data were subjected to ANOVA using the PROC MIXED procedure, with age (adult or aged), diet (0, 2.5% or 5.0% inulin inclusion), sex (male or female), and their interactions as fixed effects, while mouse nested in the age by sex interaction was used as random effect. BW and experimental cohort were used as covariates for SCFA analysis. BW analysis also included the effect of time (weeks) as a fixed effect, and its interaction with all other fixed effects, using the repeated statement and auto regressive 1 as covariance structure. The Kenward-Roger statement was used for computing the denominator degrees of freedom in all models. Normality of the residuals was tested *via* PROC UNIVARIATE procedures. Data were considered significant at *P* ≤ 0.05, while tendencies were declared at *P* ≤ 0.10, using the PDIFF statement in SAS. For ease of interpretation, estimates and standard errors for Fluidigm gene expression data were properly back-transformed.

## Results

### Single-Cell Gene Expression Analysis Highlighted an Age-Related Phenotypic Shift in the Microglia Population

**FIGURE 2:**
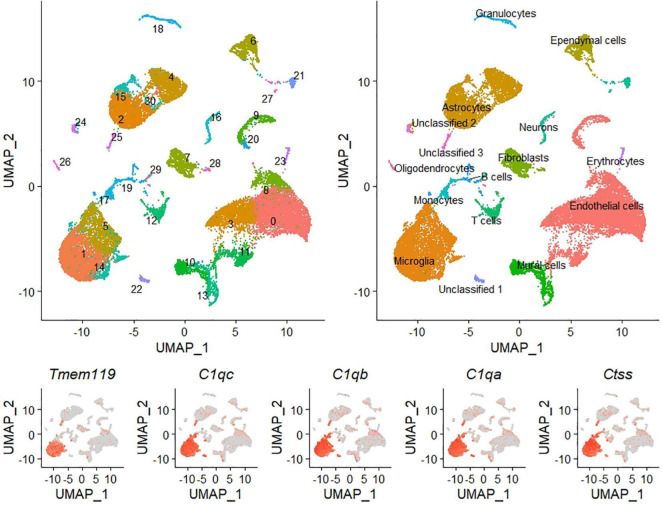
Visualization of whole brain single-cell RNA sequencing results. Uniform Manifold Approximation and Projection (UMAP) visualization of cell clusters identified during scRNA-seq analysis of mouse whole brain tissue. UMAPs includes all cells from the four samples analyzed. The top left panel identifies each cluster by number and color, and the top right panel identifies each cell type by name and color. Bottom panels illustrate the expression of five canonical microglia markers that helped identify clusters 1, 5, and 14 as microglial cells. All microglia clusters show expression of these five genes. In addition, very few cells outside the microglia grouping express any of the genes.

**FIGURE 3:**
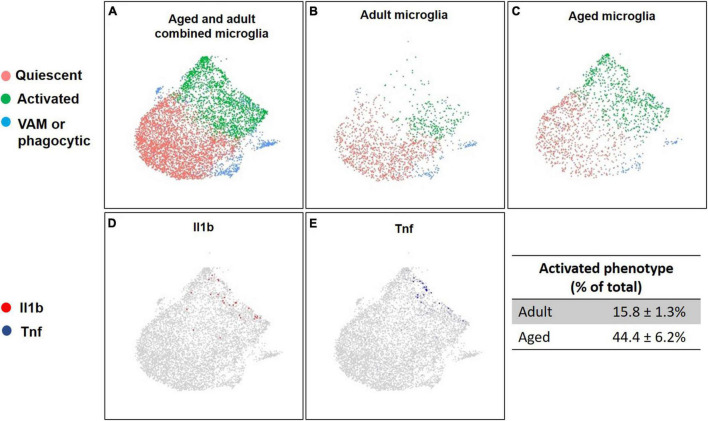
Distinct clusters of microglia and their gene expression patterns across age. Uniform Manifold Approximation and Projection (UMAP) of microglia isolated from the whole brain of adult and aged mice. Panel **A** represents the total population of microglia subdivided in three clusters identified across both groups. Microglia clusters were identified as quiescent (red), activated (green), or vascular associated (VAM) or phagocytic microglia (blue). Panels **B,C** illustrate how microglial activation states vary across age. Old mice (26 months) **(C)** have a larger pro-inflammatory activated population (green), compared to adult mice (5.25 months) **(B)**. Panels **D,E** highlight the cells expressing pro-inflammatory markers *Il1b* (red, **D**) and *Tnf* (blue, **E**).

Single-cell RNA-sequencing identified an average of 9,219 ± 990 cells per sample with a total of 55,573 unique transcripts. After removing doublets and dead cells there was an average of 7,206 ± 844 cells per sample, 2,605 features (i.e., genes), and 7,296 unique molecular identifiers per cell (see Supplementary Figures 1–4, for quality control pre- and post-filtering). Clustering analysis revealed 31 clusters, which were annotated to 13 unique cell types, and three unclassified clusters (Figure 2 and Supplementary Figure 5 for QC by cluster). Each unique cell type did not necessarily yield one cluster, as high gene expression variation between cells of the same type resulted in segregation. Considering all samples combined the most abundant cell types were endothelial cells, microglia, and astrocytes, with 10,356, 6,365, and 5,145 cells, respectively. Within the microglia population, the clustering analysis revealed three distinct microglial cell clusters that were classified by differential expression analysis of their unique gene markers (see Supplementary Figure 6) as quiescent (cluster 1; 3,810 total cells), activated (cluster 5; 1,929 total cells) and either vascular associated microglia, or phagocytic microglia (cluster 14; 626 total cells). The quiescent and activated clusters had an inverse expression of pro-inflammatory genes among their top 10 discriminatory markers, with cluster 5 having high expression of *Apoe*, *Ccl4*, *Spp1*, *Ccl3*, *Lyz2*, *Cst7*, and *Fos*, among them, while cluster 1 displayed similar downregulated fold changes for these genes. Uniform Manifold Approximation and Projection (UMAP) analysis of microglial cells showed a shift in the percentage of activated microglia between adult and aged animals. Adults had 15.8 ± 1.3% of all microglia cells in the activated cluster, compared to 44.4 ± 6.2% for their aged counterpart (Figure 3).

**FIGURE 4:**
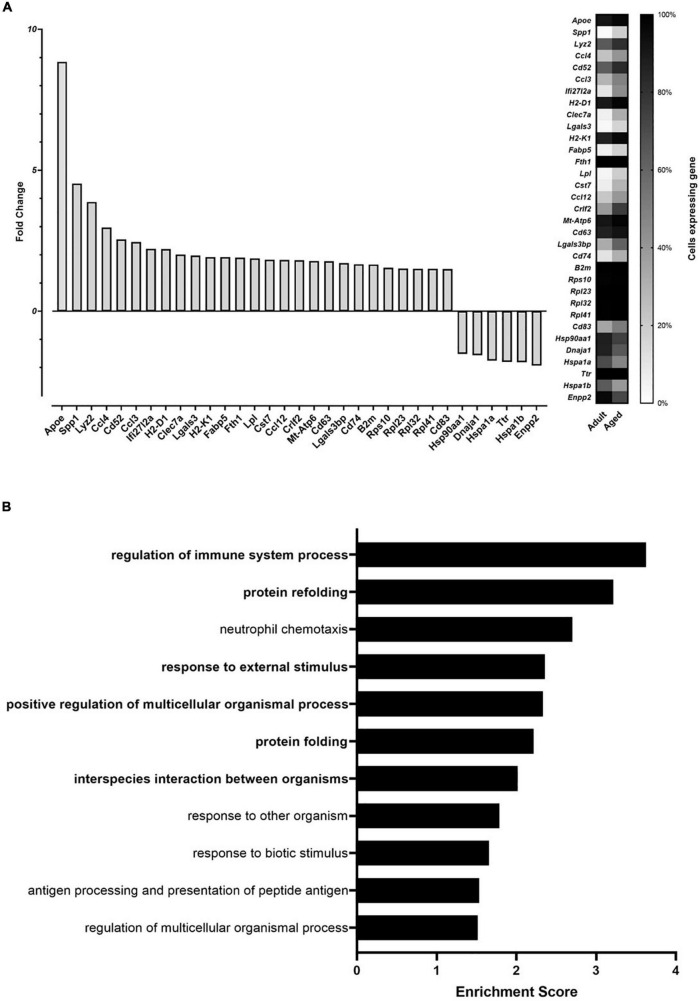
Age effect on whole brain microglia gene expression **(A)** and enrichment analysis of age affected genes **(B)**, identified *via* single cell RNA sequencing. The analysis was conducted on single cells of microglia from aged (26 months) and adult mice (5.25 months). **(A)** The left panel (bars) depicts the fold change expression in aged mice when compared to adult mice when the gene was detected. The right panel (heatmap) reports the percentage of microglia cells in which each differentially expressed gene was detected. **(B)** Gene Ontology (GO) enriched biological functions identified by enrichment analysis of the differentially expressed genes in aged compared to adult mice. Enrichment score is calculated as –log_10_ *P*-value. Higher hierarchy GO biological functions are highlighted in bold.

Single-cell gene expression analysis was insensitive to effects of fiber, but analysis of aged compared to adult brain microglia (clusters 1, 5, and 14 combined) identified 33 differentially expressed genes in aged microglia: 27 genes were upregulated and 6 were downregulated (Figure 4A). Gene enrichment analysis (Figure 4B) identified “regulation of immune system,” “protein folding and refolding,” “response to external stimulus,” and “interspecies interaction between organisms” as high hierarchy processes affected by aging (*P* < 0.01). Lower hierarchy processes (e.g., subcategories of the higher one) included immune related functions, such as “neutrophils chemotaxis,” “response to other organisms and biotic stimuli,” and “antigen processing and presentation.” Additional information regarding the enriched GO biological functions can be found in Table 1.

**TABLE 1:** Detailed results of the Gene Ontology (GO) enrichment analysis of the identified differentially expressed genes (DEG) in microglia from aged (∼2 years old) compared to adult (3–4 months old) mice.

Go term	Description	Background	DEG	Fold enrichment	Corrected *P*-value	DEG list
GO:0051239	Regulation of multicellular organismal process	2,764	18	2.96	0.0304	*Apoe↑, B2m↑, Ccl3↑, Ccl4↑, Cd63↑, Cd74↑, Cd83↑, Clec7a↑, Crlf2↑, Cst7↑, Enpp2*↓, *Fabp5↑, H2-D1↑, H2-K1↑, Hsp90aa1*↓, *Lgals3↑, Lpl↑, Spp1↑*
GO:0009605	Response to external stimulus	1,625	15	4.2	0.0044	*Apoe↑, B2m↑, Ccl12↑, Ccl3↑, Ccl4↑, Cd52↑, Cd63↑, Clec7a↑, Enpp2*↓, *H2-K1↑, Ifi27l2a↑, Lgals3↑, Lpl↑, Lyz2↑, Spp1↑*
GO:0051240	Positive regulation of multicellular organismal process	1,632	15	4.18	0.00465	*Apoe↑, B2m↑, Ccl3↑, Ccl4↑, Cd74↑, Cd83↑, Clec7a↑, Crlf2↑, Cst7↑, Enpp2*↓, *Fabp5↑, Hsp90aa1*↓, *Lgals3↑, Lpl↑, Spp1↑*
GO:0002682	Regulation of immune system process	1,096	14	5.81	0.000237	*Apoe↑, B2m↑, Ccl12↑, Ccl3↑, Ccl4↑, Cd74↑, Cd83↑, Clec7a↑, Crlf2↑, Cst7↑, H2-D1↑, H2-K1↑, Hsp90aa1*↓, *Lgals3↑*
GO:0044419	Interspecies interaction between organisms	1,040	12	5.25	0.00962	*Apoe↑, B2m↑, Ccl12↑, Ccl3↑, Ccl4↑, Cd52↑, Clec7a↑, H2-K1↑, Ifi27l2a↑, Lgals3↑, Lpl↑, Lyz2↑*
GO:0009607	Response to biotic stimulus	920	11	5.44	0.022	*B2m↑, Ccl12↑, Ccl3↑, Ccl4↑, Cd52↑, Clec7a↑, H2-K1↑, Ifi27l2a↑, Lgals3↑, Lpl↑, Lyz2↑*
GO:0051707	Response to other organism	892	11	5.61	0.0163	*B2m↑, Ccl12↑, Ccl3↑, Ccl4↑, Cd52↑, Clec7a↑, H2-K1↑, Ifi27l2a↑, Lgals3↑, Lpl↑, Lyz2↑*
GO:0006457	Protein folding	142	6	19.22	0.00609	*B2m↑, Cd74↑, Dnaja1*↓, *Hsp90aa1*↓, *Hspa1a*↓, *Hspa1b*↓
GO:0030593	Neutrophil chemotaxis	58	5	39.21	0.00199	*Ccl12↑, Ccl3↑, Ccl4↑, Lgals3↑, Spp1↑*
GO:0042026	Protein refolding	15	4	>100	0.000607	*B2m↑, Hsp90aa1*↓, *Hspa1a*↓, *Hspa1b*↓
GO:0048002	Antigen processing and presentation of peptide antigen	44	4	41.35	0.0292	*B2m↑, Cd74↑, H2-D1↑, H2-K1↑*

*For each statistically enriched GO term, we reported the total number of genes identified by the scRNA-seq analysis, the number and list (including direction of change, ↑ upregulate, ↓ downregulated by age) of DEG, fold enrichment, and the Bonferroni corrected P-value used to calculate the enrichment score reported in Figure 4.*

### Dietary Inulin Increased Cecal Short-Chain Fatty Acids

No effect of diet was detected for BW, while age, sex, and time strongly (*P* < 0.0001) affected it. Aged animals and males exhibited a higher weight than young or female mice. BW gradually increased over time, due to growth of adult males, while adult females, and aged animals (both sexes) maintained a stable weight throughout the 8 weeks on the experimental diets (see Supplementary Figure 7).

Age affected the concentration of acetate and total SCFA, with adults having higher concentrations than their aged counterparts (Table 2). Dietary inulin increased butyrate (*P* < 0.0001) and total SCFA (*P* = 0.004) in the cecum of both adult and aged animals (Table 2). There were no significant differences between 2.5 and 5% inulin. Moreover, aged mice exhibited lower levels of acetate than adult mice on the control diet and, when fed inulin, aged mice had acetate concentration similar to that observed for adults receiving no inulin. Sex also affected (*P* < 0.05) cecal concentration of butyrate, acetate, and total SCFA, as overall higher concentrations were detected in males compared to females (Table 2).

**TABLE 2:** Cecal concentration of SCFAs acetate, propionate, butyrate, and their sum, corrected for cecal water content [dry matter (DM)], in response to age (A), sex (S), or diet (D) (inulin; % w/w).

	A			S			D				*P*-value						
						
Mmol/g DM	Adult	Aged	SEM	F	M	SEM	0%	2.5%	5%	SEM	A	D	A × D	S	S × A	S × D	S × A × D
Acetate	167.4^a^	121.0^b^	10.3	129.5^a^	158.9^b^	9.3	134.8	153.1	144.8	10.7	0.006	0.40	0.57	0.04	0.39	0.95	0.66
Propionate	32.6	30.6	2.4	31.4	31.8	2.2	27.5	33.7	33.6	2.6	0.62	0.07	0.49	0.90	0.72	0.09	0.10
Butyrate	54.4	44.1	4.1	43.6^a^	55.0^b^	3.7	32.4^a^	56.5^b^	58.9^b^	4.2	0.11	<0.0001	0.61	0.04	0.31	0.12	0.97
Total	244.9^a^	191.8^b^	13.1	191.9^a^	244.7^b^	11.8	186.2^a^	241.6^b^	227.2^b^	13.6	0.01	0.004	0.13	0.004	0.64	0.73	0.20

*For each main effect the largest standard error of the mean (SEM) is reported. Different superscripts denote statistical difference of the means (P < 0.05) within each effect (A, S, and D). Treatments comprised the 2 × 3 × 2 factorial arrangement of age, diet, and sex. Data from 136 mice are included (adult, n = 63 and aged, n = 73; inulin at 0%, n = 57, 2.5%, n = 36; and 5%, n = 43; male, n = 74 and female, n = 62).*

### Dietary Inulin Inhibited Many Effects of Aging on Gene Expression in Microglia

Supplementary Table 2 shows the main effects of age (adult and aged), sex (male and female), and diet (0, 2.5, and 5.0% inulin) as well as interactions on microglial expression of genes related to aging, inflammation, and the sensome. However, because 2.5% inulin elicited maximal effects on SCFA (Table 2) and yielded more consistent results on gene expression (Supplementary Table 2) suggesting 5.0% inulin may have been more than optimal, a secondary analysis was done excluding the 5.0% inulin treatment.

#### Aging Markers

**FIGURE 5:**
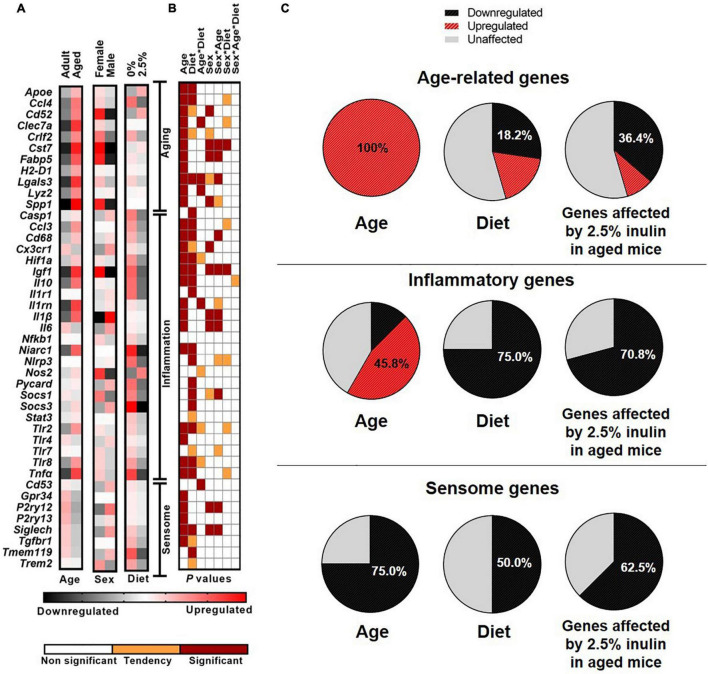
The effect of age, sex, and dietary fiber on microglial gene expression. Heat map visualization of relative expression of genes in microglia analyzed by Fluidigm from adult and aged, male and female mice fed inulin (0 or 2.5%). **(A)** The heat map represents the relative mRNA abundance of markers of aging, inflammation, and the microglial sensome with black for downregulation and red for upregulation. Each square represents the mean of *n* = 17–37 depending on the group. Treatments comprised the 2 × 2 × 2 factorial arrangement of age, diet, and sex. Data from 90 mice are included (adult, *n* = 17, and aged, *n* = 32; inulin at 0%, *n* = 25, and 2.5%, *n* = 37; male, *n* = 30 and female, *n* = 32). **(B)** The panel visually represents the *P*-value of main effects and their interaction obtained *via* ANOVA. White represents no effect (*P* > 0.10), orange indicates a tendency (0.05 < *P* ≤ 0.10), and maroon indicates statistical significance (*P* ≤ 0.05). **(C)** The pie charts represent the percentage of the targeted genes affected by age (left), diet (intermediate), or diet only on aged brain (right) with black for downregulation and red for upregulation.

Markers of aging (e.g., genes upregulated in aged individuals compared to adults) were chosen from the available literature and the scRNA-seq analysis. In primary microglia isolated from aged mice, aging markers were increased (*P* < 0.001; Figure 5). A significant (*P* < 0.05) effect of diet was detected for *Apoe*, *Ccl4*, and *Lgals3*. Specifically, inulin increased expression of *Apoe*, and decreased expression of *Ccl4* and *Lgals3*. An interaction of age and diet was detected for *Clec7a* (*P* = 0.04), *Lgals3* (*P* = 0.02), and *Lys2* (*P* = 0.04). No dietary effect on the expression of *Lgals3* was observed in adults, but inulin reduced its abundance in aged individuals.

Sex differences were evident in aging-related genes, with females displaying a generally higher (*P* < 0.05) expression of most of these markers, except *Apoe*, *Ccl4*, *H2-D1*, and *Lyz2*, for which no sex effect was observed (Figure 5). A tendency for higher expression in females compared to males, was also observed for *Crlf2* (*P* = 0.09) and *Lgals3* (*P* = 0.05). For *Cst7*, *Fabp5*, and *Lgals3* an interaction of sex and age was detected (*P* ≤ 0.01), suggesting that the higher overall expression in females was due to increased expression in aged, but not adult females. Both sexes of aged animals had higher *Lyz2* expression than adults, however the lowest expression was observed in the adult females. No other interactions were detected.

#### Inflammation

To assess the inflammatory status of microglia we measured the expression of genes encoding for cytokines, chemokines, antimicrobial proteins, sensing receptors, inflammatory signal transductors, proteins of the inflammasome, and markers of pro-inflammatory activation. As demonstrated by the scRNA-seq data, age significantly altered the expression of many inflammatory genes (Figure 5). Aged animals displayed higher (*P* < 0.001) mRNA abundance for *Casp1, Ccl3*, *Cd68*, *Hif1a*, *Igf1*, *Il10*, *Il1b*, *Il1rn*, *Niarc1*, *Tlr2*, *Tlr8*, and *Tnfa* compared to adults. At the same time, aging reduced the expression of *Cx3cr1*, *Il6*, *Socs1*, and *Tlr4* (*P* < 0.001). Independently of age, inclusion of inulin reduced (*P* < 0.05) expression of *Casp1*, *Ccl3*, *Cd68*, *Hifl1*, *Igf1*, *Il10*, *Il1r1*, *Niarc1*, *Nlrp3*, *Pycard*, *Socs1, Socs3*, *Tlr8*, and *Tnfa* (Figure 5). An interaction of age and diet was detected for *Il1rn* (*P* = 0.03), and tendencies were observed for *Hifla* (*P* = 0.07), *Nos* (*P* = 0.08) *Tlr2* (*P* = 0.06), and *Tlr8* (*P* = 0.07), as inulin inclusion was able to reduce microglia expression of these markers in aged and not in adult animals. Also, inflammation markers were affected by sex, as there were several sex by age interactions. For *Cd68*, *Igf1*, *Il1b*, *Il6*, and *Socs1*, no sex-related morphisms were observed in adult animals, while differences were noted between sexes among aged mice. Aged females displayed higher microglial expression for *Igf1*, while they had lower expression of *Cx3cr1*, *Il1b*, and *Il6*, compared to aged males.

#### Sensome

As neuroimmune sentinels, microglia continuously sense changes in the brain environment and respond to invading pathogens, toxins and cellular debris (27). For this purpose, microglia constitutively express a unique cluster of transcripts encoding proteins for sensing endogenous ligands and microbes, referred to as the sensome. Age downregulated (*P* ≤ 0.01) five out of eight targeted sensome genes (*Gpr34, P2ry12, P2ry13, Siglech*, and *Tgfbr1*), compared to their expression in adult (Figure 5). Dietary inulin altered the sensome-related gene expression of *Siglech* and *Tmem119*, with a tendency for *Tgfbr1* (*P* = 0.07). Sex effects were also observed for *P2ry12* (*P* = 0.02) and *Siglech* (*P* = 0.01), with overall female expression levels lower than males.

### Dietary Inulin Decreased *ex vivo* Microglial Tumor Necrosis Factor-Alpha Secretion in Aged Mice

**FIGURE 6:**
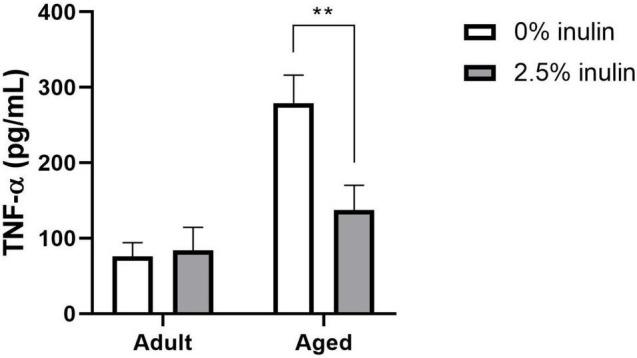
The effect of age and fiber on microglial TNF-α secretion. Microglia from aged and adult male mice fed 0 or 2.5% inulin were isolated and cultured for 36 h (*n* = 4). TNF-α concentration (pg/mL) in cell culture supernatants was assayed by ELISA. Statistically different between diets: **P < 0.01.

The age-related increase in *Tnfa* and *Il10* gene expression was reduced by dietary inulin (Figure 5). To determine if TNF-α and IL-10 protein were similarly affected, supernatants from microglia cultures were assayed. Consistent with *Tnfa* mRNA expression, microglia from aged mice spontaneously secreted more TNF-α (*P* = 0.0004; Figure 6) than microglia from adults—an effect that was blocked by feeding aged mice inulin (*P* = 0.01). IL-10 concentration was below the assay sensitivity (data not shown).

## Discussion

The results showed that microglia from adult and aged mice segregated into distinct phenotypes according to their gene expression profile. In aged mice, a considerably greater proportion of the population of microglia were identified being “activated” and a considerably smaller proportion were identified being “quiescent.” These findings, using whole brain scRNA-seq and a limited number of mice, were largely corroborated using highly purified microglia from a large cohort of mice and Fluidigm analysis to assess a selected panel of targeted genes. In the later cohort, we tested the hypothesis that increased dietary soluble fiber could counterbalance the age-related change in microglia gene expression. This is reasonable because dietary fiber affects the gut microbiota and is fermented in the colon to yield bioactive SCFAs, including butyrate. Accordingly, feeding aged mice for 8 weeks a diet with 2.5% inulin increased colonic SCFAs and mostly restored microglial cell gene expression to that seen in adults. Feeding 5.0% inulin did not further increase SCFAs and effects on microglia gene expression were by-and-large similar but more variable. Eating too much fiber can inhibit absorption of key nutrients (28) and cause gastrointestinal distress (29). Given the present findings, 5.0% inulin may have approached the tolerable upper intake level for some animals.

Our results on the effects of aging on microglia gene expression are consistent with a recent study of the different transcriptional states of microglia throughout aging (30). Using scRNA-seq with purified microglia, Hammond et al. (30) reported two microglia clusters being enriched in aging mice, as well as a third cluster comprised of macrophages and monocytes. Noteworthy was their finding that one cluster of microglia that expanded with age, expressed inflammatory genes not normally expressed by other populations of microglia. Similarly, the present study revealed that a cluster of microglia expressing inflammatory signals was enriched by aging. This subpopulation of microglia likely contributes to normal age-related neuroinflammation. Therefore, understanding the heterogeneity of microglia throughout aging and how these cells subdivide in clusters with varied functions may be important for developing intervention strategies to regulate neuroinflammation. A limitation of our scRNA-seq analysis is that it included only male mice fed 0 or 5% inulin. Therefore, from the scRNA-seq study we cannot draw conclusion regarding differences between males and females or different levels of dietary fiber.

Our study on dietary fiber that focused on expression of selected aging-, inflammatory-, and sensome-related genes in purified microglia confirmed age-related neuroinflammation. The broad impact of dietary fiber on age-related neuroinflammation was highlighted in microglia by reduced expression of genes related to the chemokine system, inflammasome, activation, and reactive status (Figure 5), as well as by reduced secretion of the inflammatory marker TNF-α (Figure 6). Indeed, nearly half of the inflammatory genes measured were upregulated in microglia due to aging, and inclusion of 2.5% dietary inulin downregulated three quarters of them (Figure 5). Expression of many anti-inflammatory genes was also reduced by inulin. These changes likely do not impair the immune response, but simply reflect a lower pro-inflammatory environment, less dependent on anti-inflammatory signals. Finally, the expression of all aged-related genes studied was upregulated in microglia of aged mice, but 2.5% dietary inulin downregulated several of them. The reduced expression of aging markers *Ccl4* and *Lgals3* due to dietary inulin is consistent with the reduced signs of neuroinflammation (30).

The mechanism by which dietary fiber can regulate microglial gene expression in the aged brain warrants investigation, but most evidence points to a mechanism dependent upon SCFA production. For example, germ-free mice had immature, malformed, and dysfunctional microglia indicating an important role for the microbiota in microglial maturation and function (12). Reconstitution of a diverse intestinal microbiota or providing a mix of SCFAs (acetate, propionate, and butyrate) in drinking water normalized microglia after 4 weeks (12), indicating a lack of microbial-derived SCFAs in germ-free mice was responsible for aberrant microglia. The SCFAs acetate, propionate and butyrate are produced by microbiota in the colon and distal small intestine by fermentation of resistant starch, dietary fiber, other low-digestible polysaccharides and host mucin oligosaccharides. Noteworthy in the present study is the finding that aged mice had lower total SCFAs in the cecum and that dietary fiber significantly increased SCFAs. A “control” diet containing 1% cellulose was compared to a diet containing 1% cellulose *and* 2.5% inulin. Inulin is a plant-derived fermentable fiber that is a substrate for production of SCFAs (31). In aged mice, acetate and total SCFAs were significantly lower and there was a tendency for lower butyrate (*P* = 0.09). Dietary inulin (2.5%) resulted in a 60% increase in butyrate, a 15% increase in acetate, and a 30% increase in total SCFAs. This may be important because butyrate has anti-inflammatory properties and it and other SCFA concentrations may have been less than optimal in aged mice. We did a correlation analysis to assess the relationship between SCFA concentration (acetate, butyrate, propionate, and total SCFA) and gene expression in microglia from aged mice (Supplementary Table 3). Although the correlation coefficients were low, we found higher butyrate to be associated with reduced expression of several age-related (*Fabp5* and *Lgals 3*) and inflammatory (*Cd68, Igf1, Niarc1, Socs1*, and *Tlr8*) genes (*P* < 0.05), as well as a tendency (*P* < 0.1) for other inflammatory genes (*HIF1a, Il1rn*, and *Tnf*). All the SCFA alone or as total SCFA presented significant correlation with reduced expression of the inflammatory genes *Cd68* and *Igf1*. These findings suggest differences in the intestinal microbiota between adult and aged mice, even in a highly controlled setting; and that the intestinal microbiota in aged mice remained responsive to dietary fiber to a point. This is important because inadequate intake of dietary fiber is common in the elderly (17).

In support of an effect of aging on gut microbiota and SCFA production, Spychala et al. (32) reported reduced SCFA levels (acetate and propionate) in both young and aged mice harboring aged microbiota following fecal transplant gavage (FTG). Furthermore, Lee et al. (33) reported durable differences in bacterial β diversity in germ-free mice receiving FTG from young or aged mice. FTG from young mice yielded a microbiome more abundant in SCFA-producing taxa and higher levels of acetate, propionate, and butyrate. In addition, FTG from aged mice was associated with signs of cognitive aging including depressive-like behavior, impaired short-term memory, and impaired spatial memory.

Short-chain fatty acids are taken up by enterocytes of the intestinal mucosa *via* monocarboxylate transporter 1 and can be detected in hepatic portal blood (34, 35). SCFAs provide energy for enterocytes and maintain integrity of the gut epithelium, but by signaling through free fatty acid receptor (FFAR)2 and FFAR3 (i.e., GPR43 and GPR41, respectively) also regulate diverse physiologic systems. For example, FFAR2 is expressed by intestinal epithelial cells, including enteroendocrine cells, and partially regulates Peptide YY expression (36); and on enteric leukocytes is important for resolution of intestinal inflammation (37). Consistent with this notion, in a recent study we reported aged mice fed a low fiber diet exhibited histological signs of inflammation in the distal colon associated with immune cell infiltration in the lamina propria—an outcome not seen in aged animals fed a high fiber diet or adults on a low or high fiber diet (38). Interestingly, in a study of how aging affected vagal afferent signaling to the brain during peripheral infection, we reported that aged mice showed a marked increase in c-Fos expression in brain areas receiving input from the vagus nerve, including the nucleus of the solitary tract and area postrema, after intraperitoneal injection of lipopolysaccharide (39). Therefore, in aging, intestinal inflammation due to decreased SCFAs may increase viscerosensory signaling to influence brain microglia. Adding complexity, FFAR3 is expressed by peripheral nerves of the hepatic portal vein (40, 41) and studies in a transgenic reporter mouse expressing red fluorescent protein under the control of the FFAR3 promoter revealed the presence of FFAR3 on neurons of the submucosal and myenteric layers of the gut (41) and in sensory ganglia of the vagus nerve (42), thus providing a mechanism for intestinal microbiota to regulate afferent signaling to the brain and its microglia. Clearly, identification of the communication pathway(s) enabling dietary fiber to affect brain microglia requires more research.

A final point to highlight is the observed differences between males and females. Aged females had lower acetate, butyrate, and total SCFA concentrations than aged males. This is aligned with the baseline values of SCFAs reported by El-Hakim et al. (43), with males showing higher values than females. Consistent with the idea of SCFAs effects on microglial cell gene expression, on the one hand, females with lower SCFAs had higher expression of several genes associated with aging (e.g., *Cd52, Cst7, Fabp5*, and *Spp1*) and inflammation (e.g., *Igf1 and Socs1*). On the other hand, aged females compared to aged males had lower expression of sensome-related genes (e.g., *P2ry12* and *Siglech*). Collectively, these results are consistent with another study demonstrating up-regulation of genes related to inflammation and immune components in the female brain compared to male (44). Thion et al. (45) also reported female microglia displayed higher expression of inflammatory genes compared to male microglia, revealed by GO enrichment analysis from RNA-seq data. Whether this underlies increased susceptibility of females to neurological diseases, like Alzheimer’s disease (46), is unknown.

## Conclusion

The present results show the propensity for microglia to become pro-inflammatory during aging and that aged mice exhibit lower levels of SCFAs compared to adult mice. Dietary supplementation with high fermentable fiber may help prevent or reverse the age-related phenotypic shift of microglia since it led to higher levels of SCFAs and a less inflammatory microglial phenotype. The results further suggest that females are more vulnerable to the age-related phenotypic shift of microglia, perhaps making them more prone to develop neurological diseases. Thus, dietary fiber may be more beneficial for older females, especially since in the present study, aged females had lower concentration of SCFAs in the cecum, suggesting a possible microbial dysbiosis and reduction in fermentation capacity.

## Data Availability Statement

The datasets presented in this study can be found in online repositories. The names of the repository/repositories and accession number(s) can be found below: https://www.ncbi.nlm.nih.gov/geo/, GSE163055.

## Ethics Statement

All animal care procedures for this study (protocol 18230) were approved by the Institutional Animal Care and Use Committee of the University of Illinois and were in accordance with institutional guidelines and regulation.

## Author Contributions

RJ, JW, and AS designed and coordinated the animal trial, which was run by LR, MV-R, MC-S, NH, SW, and KS-D. MV-R performed bioinformatics statistical analysis. MV-R wrote the main draft of the manuscript, with inputs from all authors, which read and approved the final manuscript.

## Conflict of Interest

The authors declare that the research was conducted in the absence of any commercial or financial relationships that could be construed as a potential conflict of interest.

## Publisher’s Note

All claims expressed in this article are solely those of the authors and do not necessarily represent those of their affiliated organizations, or those of the publisher, the editors and the reviewers. Any product that may be evaluated in this article, or claim that may be made by its manufacturer, is not guaranteed or endorsed by the publisher.

## Footnotes

1
pantherdb.org

## Abbreviations

BW: body weight
DM: dry matter
DMEM: Dulbecco’s Modified Eagle Medium
EDTA: ethylenediaminetetraacetic acid
FFAR2: free fatty acid receptor 2
FFAR3: free fatty acid receptor 3
FTG: fecal transplant gavage
GO: gene ontology
IL-10: interleukin 10
IL-4: interleukin 4
PBS: phosphate buffered saline
SCFA: short-chain fatty acid
scRNA-seq: single-cell RNA sequencing
SIM: selected ion monitoring
TNF- α: tumor necrosis factor-alpha.
