# Supplementary material for: Dietary Fiber as a Counterbalance to Age-Related Microglial Cell Dysfunction
Source: Front Nutr. 2022 Mar 14;9:835824. doi: 10.3389/fnut.2022.835824 (PMC8964049; doi:10.3389/fnut.2022.835824)

Supplementary material Vailati-Riboni et al.

SUPPLEMENTARY TABLES

Supplementary Table S1. Primer information (Integrated DNA Technologies, Assay ID) for Fluidigm gene expression analysis.

Gene	Assay ID
Actb	Mm.PT.39a.22214843.g
Apoe	Mm.PT.58.33516165
Casp1	Mm.PT.58.10878305.g
Ccl3	Mm.PT.58.29283216
Ccl4	Mm.PT.58.5219433
Cd52	Mm.PT.58.434794115
Cd53	Mm.PT.58.30699738
Cd68	Mm.PT.58.12034788.g
Clec7a	Mm.PT.58.43348348
Crlf2	Mm.PT.58.41781142.g
Cst7	Mm.PT.58.8810317
Cx3cr1	Mm.PT.58.17555544
Fabp5	Mm.PT.58.29898197.gs
Gapdh	Mm.PT.39a.1
Gpr34	Mm.PT.58.46001700
H2-D1	Mm.PT.58.42136026.g
Hif-1α	Mm.PT.58.16742601
Ido1	Mm.PT.58.29540170
Igf1	Mm.PT.58.32726889
Il10	Mm.PT.58.23604055
Il1r1	Mm.PT.58.43781580
Il1rn	Mm.PT.58.43781580
Il1β	Mm.PT.58.41616450
Il6	Mm.PT.58.13354106
Lgals3	Mm.PT.58.29423458
Lyz2	Mm.PT.58.7139960
Nfkb1	Mm.PT.58.41505973
Niarc1	Mm.PT.58.33653569.g
Nlrp3	Mm.PT.58.13974318
Nos2	Mm.PT.58.5680554
P2ry12	Mm.PT.58.43542033
P2ry13	Mm.PT.58.42597879.g

Pycard	Mm.PT.56a.42872867
Siglech	Mm.PT.58.45915252
Socs1	Mm.PT.58.11527306.g
Socs3	Mm.PT.58.7804681
Ssp1	Mm.PT.58.43709208
Stat3	Mm.PT.58.11877007
Tgfbr1	Mm.PT.58.10230349
Tlr2	Mm.PT.58.45820113
Tlr4	Mm.PT.58.41643680
Tlr7	Mm.PT.58.10526075
Tlr8	Mm.PT.58.16021150
Tmem119	Mm.PT.58.6766267
Tnf	Mm.PT.58.12575861
Trem2	Mm.PT.58.7992121

Supplementary Table S2. Main effect of age (A; aged or adult), sex (S; female, F, or male, M), and soluble fiber dietary inclusion (D, diet; inulin; 0, 2.5, or 5%, w/w) on whole brain microglia expression of markers of aging, inflammation, and microglia sensome.

	**A**			**S**			**D**				***P-value***						
**Gene**	**Adult**	**Aged**	**SEM**	**F**	**M**	**SEM**	**0%**	**2.5%**	**5%**	**SEM**	**A**	**D**	**A*D**	**S**	**S*A**	**S*D**	**S*A*D**
Aging related																	
Apoe	0.19[b]	0.34[a]	0.02	0.27	0.24	0.02	0.22[b]	0.30[a]	0.24[ab]	0.02	<.001	0.03	0.88	0.24	0.39	0.84	0.30
Ccl4	0.53[b]	1.73[a]	0.19	1.02	0.90	0.11	1.22[a]	0.70[b]	1.04[a]	0.18	<.001	0.01	0.82	0.42	0.53	0.32	0.83
Cd52	0.37[b]	1.33[a]	0.17	0.92[a]	0.54[b]	0.12	0.74[ab]	0.89[a]	0.52[b]	0.13	<.001	0.05	0.69	0.01	0.15	0.22	0.62
Clec7a	0.20[b]	1.60[a]	0.08	0.60[a]	0.53[b]	0.03	0.57	0.55	0.55	0.04	<.001	0.95	0.02	0.07	0.01	0.09	0.45
Crlf2	0.43[b]	1.26[a]	0.18	0.90[a]	0.60[b]	0.13	0.87	0.63	0.72	0.17	<.001	0.42	0.41	0.04	0.53	0.65	0.81
Cst7	0.07[b]	0.83[a]	0.05	0.31[a]	0.18[b]	0.02	0.22	0.25	0.23	0.02	<.001	0.54	0.26	<.001	<.001	0.09	0.80
Fabp5	0.32[b]	1.64[a]	0.15	0.95[a]	0.56[b]	0.09	0.78	0.61	0.81	0.10	<.001	0.14	0.86	<.001	<.001	0.91	0.86
H2-D1	0.29[b]	0.61[a]	0.02	0.42	0.42	0.02	0.42	0.40	0.44	0.02	<.001	0.30	0.32	0.71	0.63	0.90	0.64
Lgals3	0.24[b]	1.92[a]	0.08	0.75[a]	0.62[b]	0.03	0.74[a]	0.62[b]	0.69[ab]	0.05	<.001	0.06	0.06	0.01	<.001	0.55	0.87
Lyz2	0.38[b]	1.07[a]	0.03	0.62	0.66	0.02	0.63	0.65	0.63	0.03	<.001	0.88	0.22	0.19	0.01	0.89	0.32
Ssp1	0.03[b]	1.91[a]	0.13	0.32[a]	0.18[b]	0.02	0.24	0.24	0.24	0.02	<.001	1.00	0.55	<.001	<.001	0.49	0.72
Inflammation																	
Casp1	0.28[b]	0.35[a]	0.03	0.29	0.34	0.03	0.37[a]	0.23[b]	0.36[a]	0.05	0.06	<.001	0.75	0.29	0.93	0.97	0.82
Ccl3	0.54[b]	1.20[a]	0.08	0.80	0.80	0.06	0.96[a]	0.65[b]	0.83[a]	0.09	<.001	0.01	0.52	0.97	0.78	0.20	0.67
Cd68	0.28[b]	0.46[a]	0.03	0.37	0.36	0.03	0.40[a]	0.27[b]	0.43[a]	0.04	<.001	<.001	0.64	0.80	0.01	0.13	0.35
Cx3cr1	1.23[a]	0.87[b]	0.06	0.93[b]	1.15[a]	0.06	1.06[ab]	0.92[b]	1.12[a]	0.07	<.001	0.04	0.21	<.001	0.02	0.27	0.79
Hif1a	0.33[b]	0.56[a]	0.06	0.45	0.41	0.04	0.52[a]	0.33[b]	0.46[ab]	0.07	<.001	0.02	0.06	0.48	0.39	0.95	0.96
Igf1	0.08[b]	0.69[a]	0.07	0.31[a]	0.17[b]	0.03	0.28[a]	0.15[b]	0.30[a]	0.04	<.001	<.001	0.46	<.001	<.001	0.19	0.91
Il10	0.12[b]	0.42[a]	0.06	0.20	0.24	0.04	0.27[a]	0.14[b]	0.30[a]	0.05	<.001	<.001	0.41	0.39	0.72	0.25	0.31
Il1β	0.44[b]	1.64[a]	0.11	0.65[b]	1.10[a]	0.08	0.82	0.87	0.86	0.07	<.001	0.88	0.91	<.001	0.01	0.07	0.49
Il1r1	17.4	16.9	1.80	16.0	18.3	2.00	21.6[a]	13.1[b]	17.8[a]	3.10	0.84	0.02	0.70	0.36	0.53	0.22	0.65
Il1rn	0.13[b]	0.97[a]	0.07	0.35	0.36	0.03	0.37	0.32	0.38	0.04	<.001	0.30	0.02	0.82	0.11	0.97	0.90
Il6	0.77[a]	0.51[b]	0.04	0.56[b]	0.69[a]	0.04	0.63	0.59	0.65	0.05	<.001	0.48	0.65	0.01	0.02	0.84	0.37
Nfkb1	0.75	0.81	0.09	0.84	0.72	0.09	0.89	0.71	0.75	0.13	0.66	0.47	0.30	0.30	0.44	0.23	0.76
Niarc1	0.18[b]	0.67[a]	0.08	0.36	0.34	0.04	0.50[a]	0.20[b]	0.42[a]	0.08	<.001	<.001	0.50	0.70	0.12	0.86	0.77
Nlrp3	1.30	1.22	0.11	1.24	1.29	0.11	1.59[a]	0.89[b]	1.43[a]	0.17	0.58	<.001	0.74	0.73	0.30	0.36	0.36
Nos2	0.09	0.14	0.03	0.17[a]	0.08[b]	0.03	0.10	0.16	0.10	0.04	0.14	0.27	0.16	0.01	0.94	0.27	0.77
Pycard	0.70	0.85	0.08	0.71	0.84	0.08	0.86[a]	0.55[b]	0.97[a]	0.12	0.16	<.001	0.77	0.25	0.94	0.92	0.87
Socs1	1.00[a]	0.71[b]	0.09	0.97[a]	0.73[b]	0.08	0.96[a]	0.70[b]	0.88[ab]	0.10	<.001	0.08	0.98	0.01	0.03	0.55	0.48
Socs3	0.34	0.42	0.05	0.33	0.43	0.05	0.52[a]	0.19[b]	0.55[a]	0.09	0.25	<.001	0.90	0.15	0.65	0.57	0.73

Stat3	0.83	0.99	0.07	0.93	0.88	0.07	1.04	0.84	0.86	0.11	0.12	0.24	0.60	0.62	0.67	0.34	0.65
Tlr2	0.50^{b}	1.20^{a}	0.07	0.80	0.75	0.04	0.83	0.71	0.78	0.06	<.001	0.21	0.08	0.45	0.30	0.44	0.95
Tlr4	1.06^{a}	0.80^{b}	0.07	0.88	0.96	0.06	0.94	0.86	0.97	0.08	<.001	0.41	0.66	0.33	0.49	0.33	0.87
Tlr7	0.60	0.56	0.04	0.63^{a}	0.53^{b}	0.04	0.62	0.52	0.60	0.05	0.36	0.15	0.96	0.05	0.16	0.28	0.67
Tlr8	0.30^{b}	0.65^{a}	0.04	0.45	0.43	0.03	0.50^{a}	0.35^{b}	0.49^{a}	0.04	<.001	<.001	0.21	0.48	0.02	0.46	0.57
Tnfα	0.27^{b}	1.46^{a}	0.13	0.65	0.60	0.06	0.83^{a}	0.39^{b}	0.75^{a}	0.11	<.001	<.001	0.49	0.61	0.18	0.13	0.82
Sensome																	
Cd53	0.95	0.87	0.07	0.88	0.95	0.07	0.99	0.89	0.86	0.09	0.35	0.47	0.02	0.43	0.57	0.20	0.68
Gpr34	0.52^{a}	0.33^{b}	0.04	0.42	0.40	0.03	0.41	0.38	0.45	0.04	<.001	0.39	0.68	0.64	0.61	0.70	0.40
P2ry12	1.06^{a}	0.59^{b}	0.07	0.69^{b}	0.91^{a}	0.06	0.81	0.75	0.81	0.07	<.001	0.68	0.26	<.001	<.001	0.28	0.80
P2ry13	0.64^{a}	0.36^{b}	0.05	0.48	0.48	0.04	0.46^{ab}	0.42^{b}	0.57^{a}	0.06	<.001	0.08	0.81	0.98	0.21	0.57	0.28
Siglech	0.66^{a}	0.48^{b}	0.04	0.50^{b}	0.64^{a}	0.04	0.59^{ab}	0.50^{b}	0.62^{a}	0.05	<.001	0.06	0.03	0.01	0.02	0.79	0.94
Tgfbr1	0.42^{a}	0.32^{b}	0.04	0.38	0.35	0.04	0.38^{ab}	0.30^{b}	0.44^{a}	0.06	0.04	0.04	0.13	0.55	0.42	0.68	0.48
Tmem119	0.71^{a}	0.47^{b}	0.12	0.55	0.60	0.10	0.72^{a}	0.37^{b}	0.73^{a}	0.16	0.08	0.02	0.94	0.69	0.67	0.56	0.61
Trem2	0.45	0.49	0.07	0.50	0.43	0.07	0.50^{ab}	0.33^{b}	0.62^{a}	0.11	0.67	0.02	0.38	0.49	0.36	0.63	0.57

Estimates of mRNA relative abundance and standard error of the mean (SEM) generated by the ANOVA model are reported. Data have been properly back-transformed for ease of interpretation. Different superscript indicate statistical significance at $P \leq 0.05$ for each gene within each effect (A, S, and D).

Supplementary Table S3. Correlations between SCFA corrected cecal concentration (acetate, butyrate, propionate, and total SCFA) and whole brain microglia log2 normalized mRNA abundance of target genes in aged mice, fed 0 or 2.5% inulin.

	**Acetate**		**Propionate**		**Butyrate**		**Total SCFA**	
	**r**	**p**	**r**	**p**	**r**	**p**	**r**	**p**
**Aging**								
Apoe	0.07	0.72	-0.08	0.68	-0.01	0.94	0.09	0.66
Ccl4	-0.10	0.61	-0.15	0.45	-0.07	0.71	-0.08	0.70
Cd52	-0.06	0.77	-0.20	0.33	0.05	0.78	0.06	0.75
Clec7a	-0.28	0.15	-0.33	0.10 [t]	-0.24	0.23	-0.24	0.21
Crlf2	-0.13	0.50	-0.22	0.27	-0.09	0.66	-0.10	0.62
Cst7	-0.14	0.48	-0.19	0.34	-0.33	0.09 [t]	-0.12	0.53
Fabp5	-0.25	0.19	-0.25	0.21	-0.53	0.00*	-0.25	0.20
H2.D1	-0.17	0.38	-0.06	0.78	-0.04	0.84	-0.09	0.64
Lgals3	-0.31	0.11	-0.24	0.23	-0.55	0.00*	-0.28	0.14
Lyz2	-0.13	0.50	-0.15	0.47	-0.07	0.73	-0.12	0.55
Ssp1	-0.24	0.20	-0.08	0.69	-0.27	0.16	-0.21	0.28
**Inflammation**								
Casp1	-0.28	0.14	-0.22	0.26	-0.27	0.16	-0.26	0.19
Ccl3	-0.14	0.46	-0.03	0.87	-0.10	0.60	-0.13	0.50
Cd68	-0.40	0.03*	-0.41	0.03*	-0.58	0.00*	-0.39	0.04*
Cx3cr1	-0.09	0.63	-0.14	0.48	0.13	0.50	-0.04	0.83
HIF.1a	-0.29	0.12	-0.27	0.17	-0.33	0.09 [t]	-0.23	0.25
Igf1	-0.34	0.07 [t]	-0.42	0.03*	-0.53	0.00*	-0.35	0.07 [t]
Il.10	-0.09	0.66	0.11	0.58	-0.15	0.43	-0.09	0.66
Il.1b	0.09	0.64	0.24	0.22	0.23	0.23	0.11	0.59
Il.1rn	-0.26	0.17	-0.23	0.24	-0.35	0.07 [t]	-0.20	0.30
Il.6	0.02	0.91	-0.06	0.75	0.16	0.42	0.00	0.99
Il1r1	-0.19	0.36	-0.19	0.38	-0.15	0.47	-0.17	0.41
Nfkb1	-0.11	0.59	-0.25	0.22	-0.11	0.57	-0.04	0.85
Niarc1	-0.22	0.24	-0.02	0.92	-0.42	0.03*	-0.17	0.38
Nlrp3	-0.10	0.62	-0.24	0.23	-0.11	0.56	-0.13	0.52
Nos2	0.03	0.88	-0.19	0.33	-0.16	0.43	-0.07	0.71
Pycard	-0.23	0.22	-0.25	0.21	-0.16	0.42	-0.20	0.30
Socs1	-0.06	0.76	0.02	0.91	-0.38	0.05*	-0.01	0.97
Socs3	-0.11	0.58	-0.01	0.97	-0.18	0.36	-0.13	0.50
Stat3	-0.08	0.67	-0.15	0.47	-0.16	0.42	-0.03	0.89
Tlr2	-0.24	0.22	-0.29	0.15	-0.15	0.44	-0.20	0.31
Tlr4	-0.04	0.85	-0.20	0.31	0.12	0.55	0.01	0.98
Tlr7	-0.14	0.48	-0.36	0.07 [t]	-0.16	0.43	-0.14	0.49
Tlr8	-0.28	0.14	-0.18	0.36	-0.48	0.01*	-0.32	0.10
Tnf	-0.20	0.31	-0.04	0.84	-0.36	0.06 [t]	-0.25	0.20

**Sensome**								
Cd53	-0.17	0.38	-0.23	0.24	-0.05	0.81	-0.17	0.40
Gpr34	-0.34	0.07 ^{t}	-0.43	0.02*	-0.20	0.30	-0.32	0.09 ^{t}
P2ry12	-0.16	0.40	-0.23	0.25	0.14	0.48	-0.09	0.65
P2ry13	-0.13	0.49	-0.23	0.24	0.04	0.83	-0.03	0.86
Siglech	-0.32	0.09 ^{t}	-0.28	0.16	-0.14	0.48	-0.27	0.17
Tgfbr1	-0.08	0.69	-0.28	0.16	-0.11	0.59	-0.05	0.79
Tmem119	-0.14	0.47	-0.22	0.28	-0.04	0.86	-0.09	0.64
Trem2	-0.22	0.27	-0.43	0.03*	-0.30	0.13	-0.20	0.32

*Significant P value ($P < 0.05$); ^{t} Tendency ($P < 0.1$).

SUPPLEMENTARY FIGURES

Supplementary Figure S1. Violing plot pre-filtering of total number of genes (nFeature), total number of molecules (nCount) and percentage of mitochondrial genes (percent.mt) per cell in the four samples used for 10X analysis.

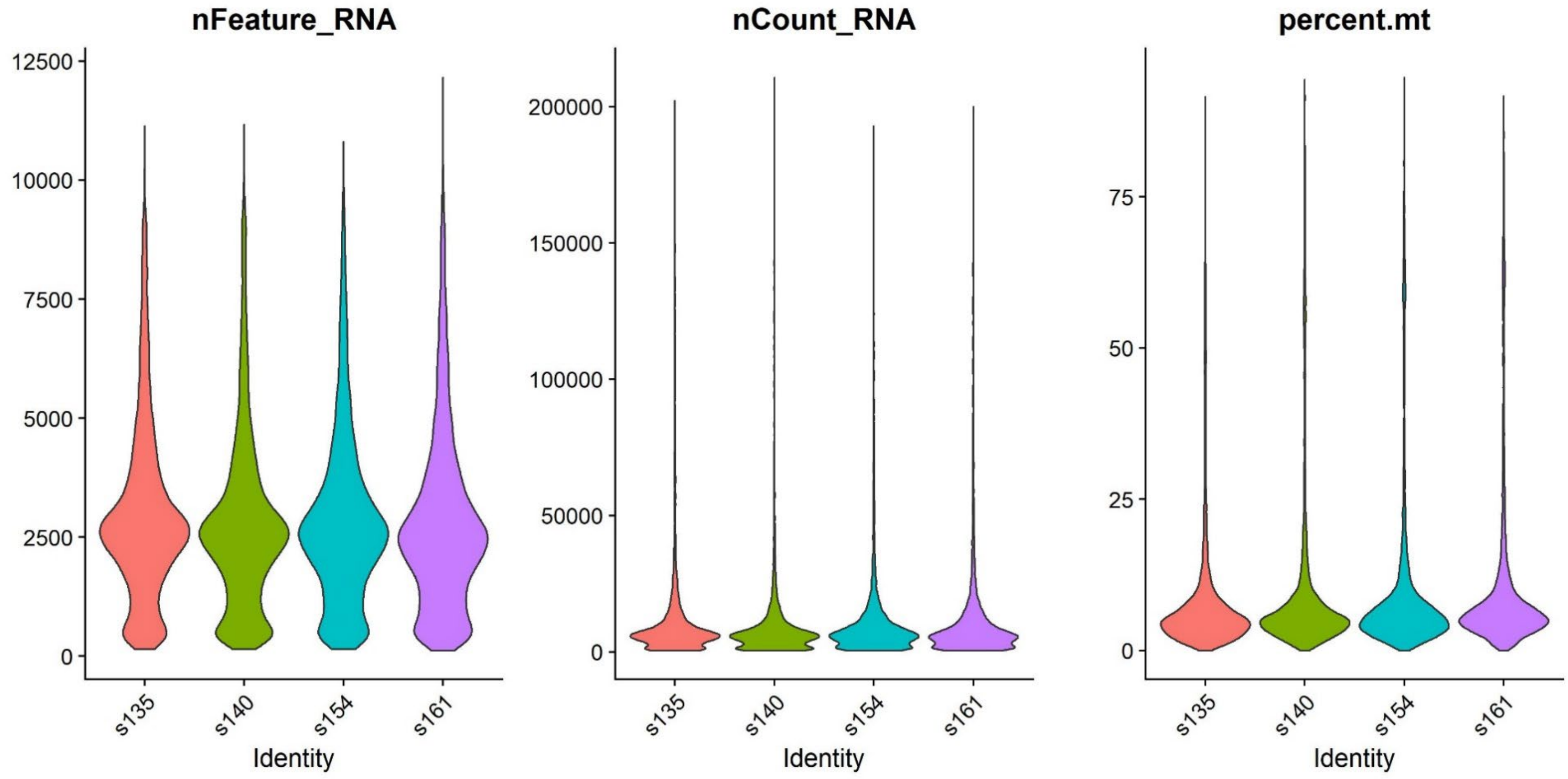

Supplementary Figure S2. Pre-filtering correlation scatter plots of total number of genes (nFeature), total number of molecules (nCount) and percentage of mitochondrial genes (percent.mt) per cell split by sample.

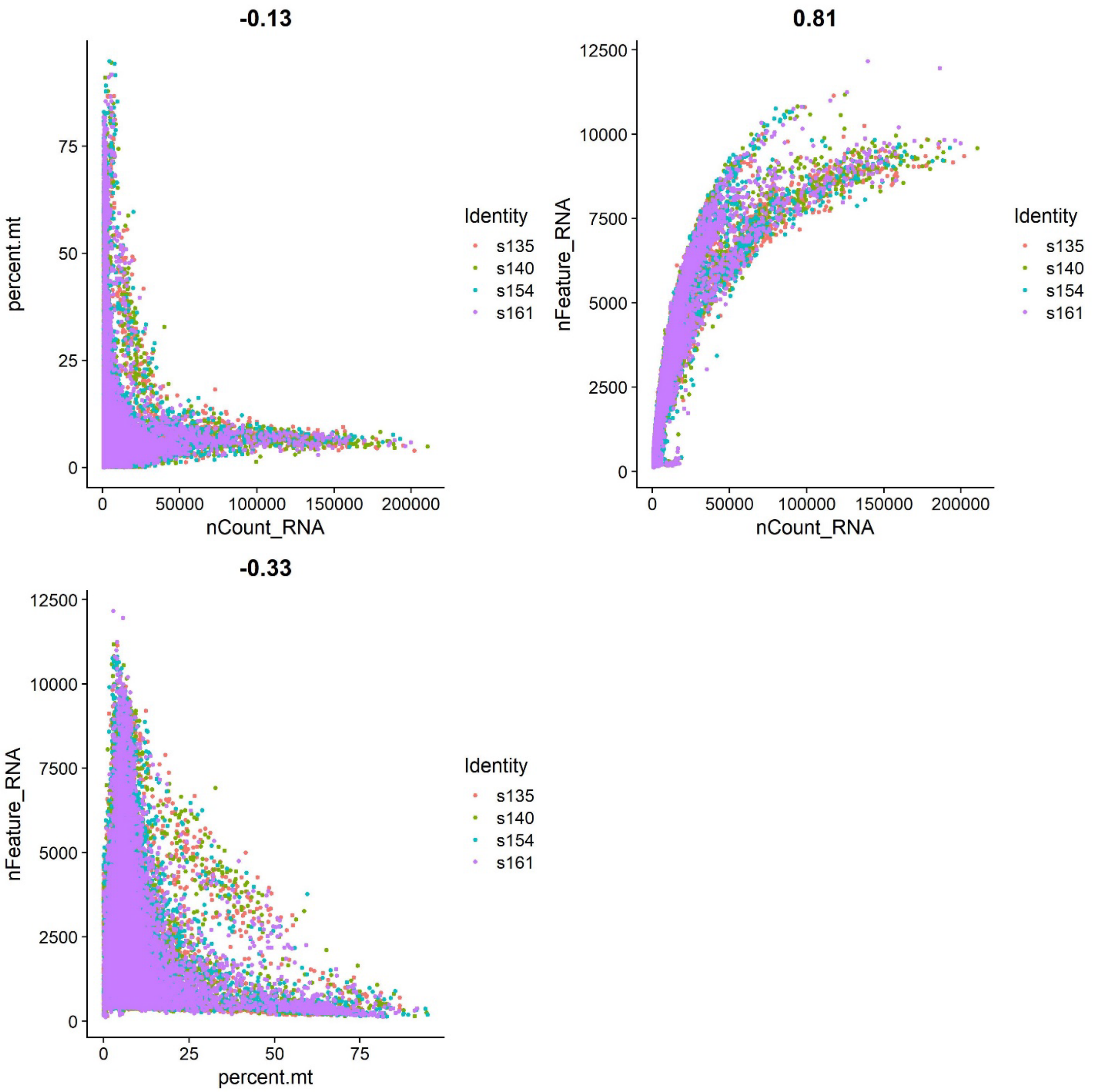

Supplementary Figure S3. Violing plot post-filtering of total number of genes (nFeature), total number of molecules (nCount) and percentage of mitochondrial genes (percent.mt) per cell in the four samples used for 10X analysis.

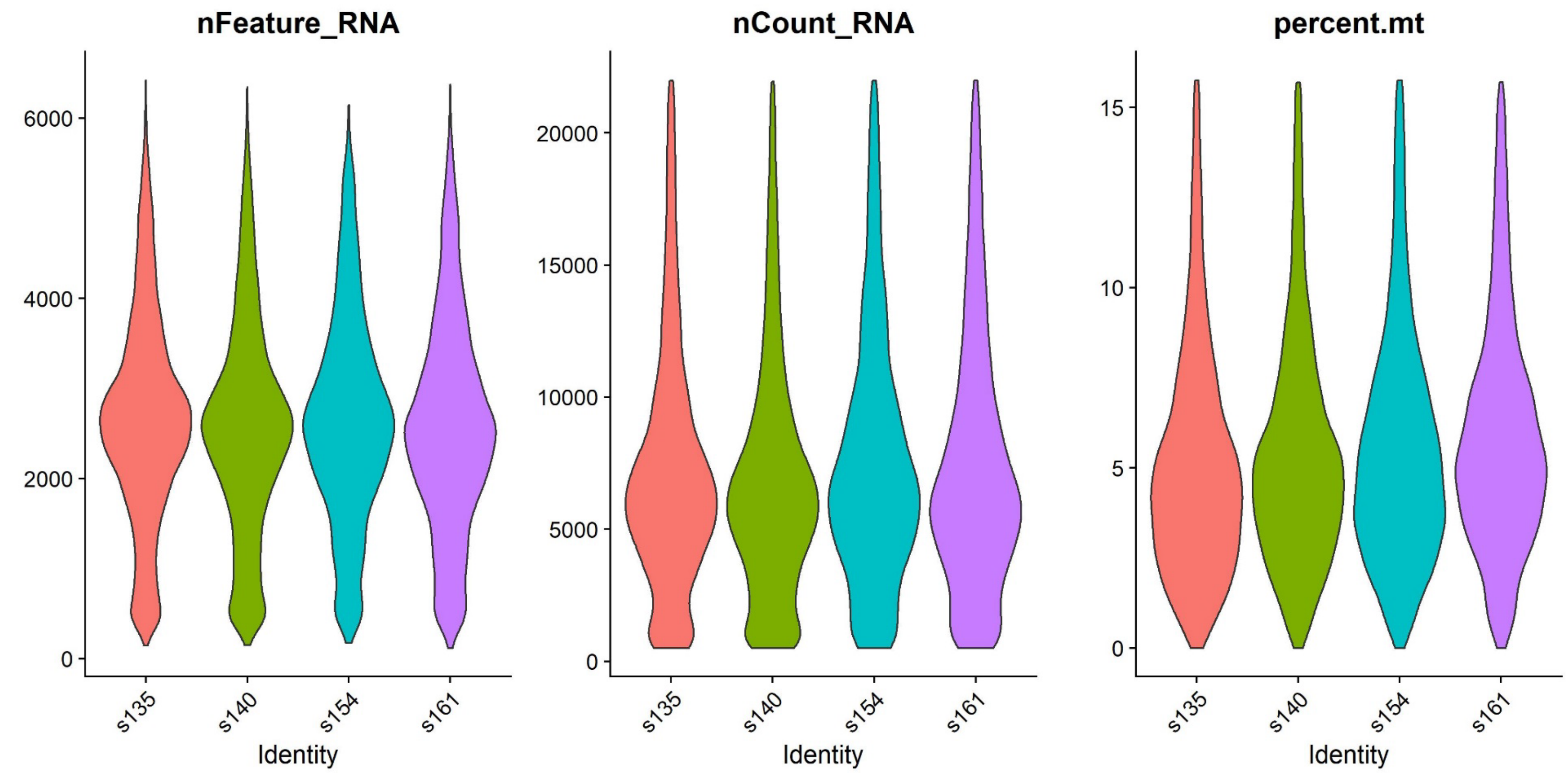

Supplementary Figure S4. Pre-filtering correlation scatter plots of total number of genes (nFeature), total number of molecules (nCount) and percentage of mitochondrial genes (percent.mt) per cell split by sample.

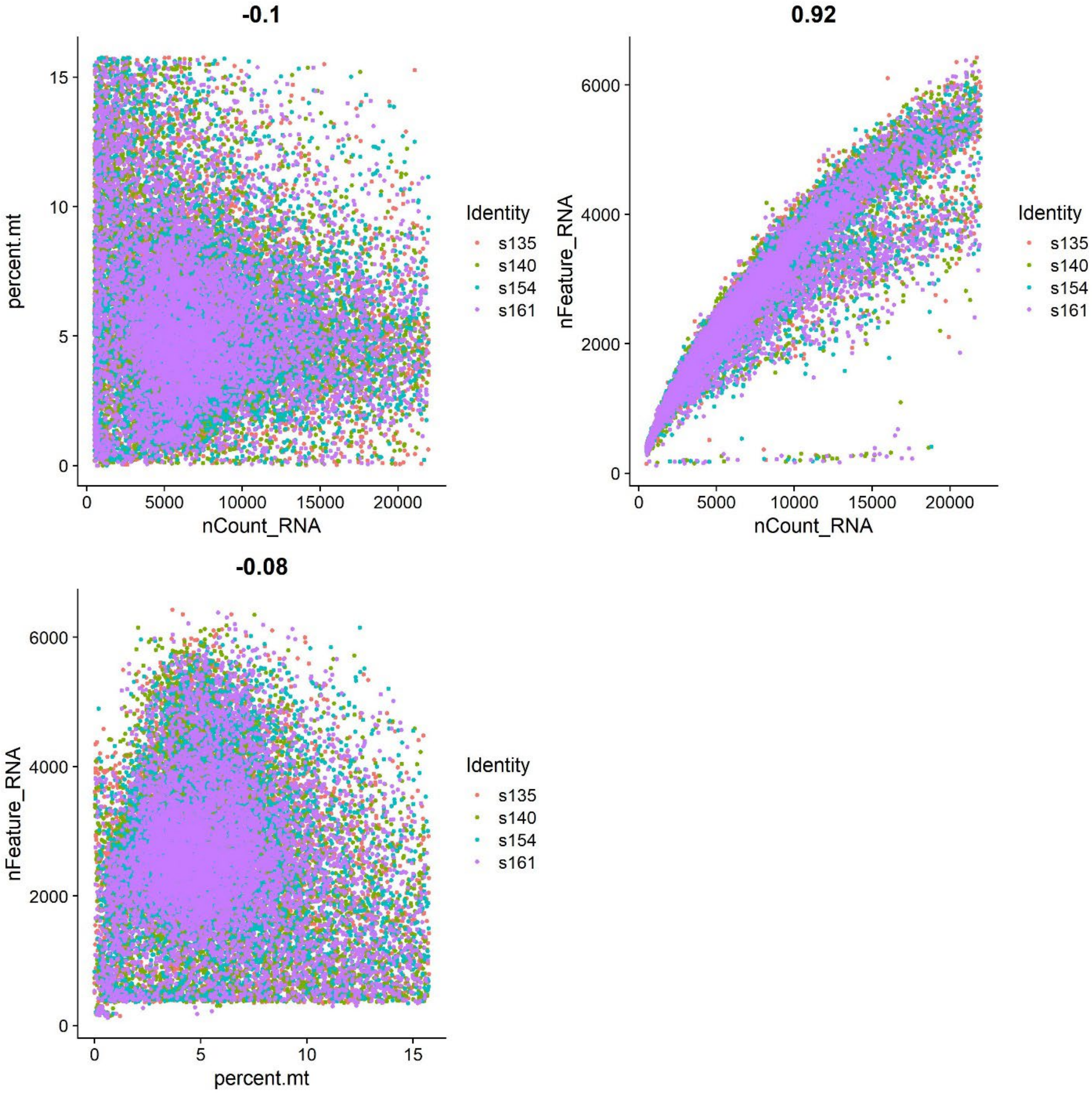

Supplementary Figure S5. Violing plot post-filtering of total number of genes (nFeature), total number of molecules (nCount) and percentage of mitochondrial genes (percent.mt) per cell by cluster. UMAP representation of identify clusters is reported on the left.

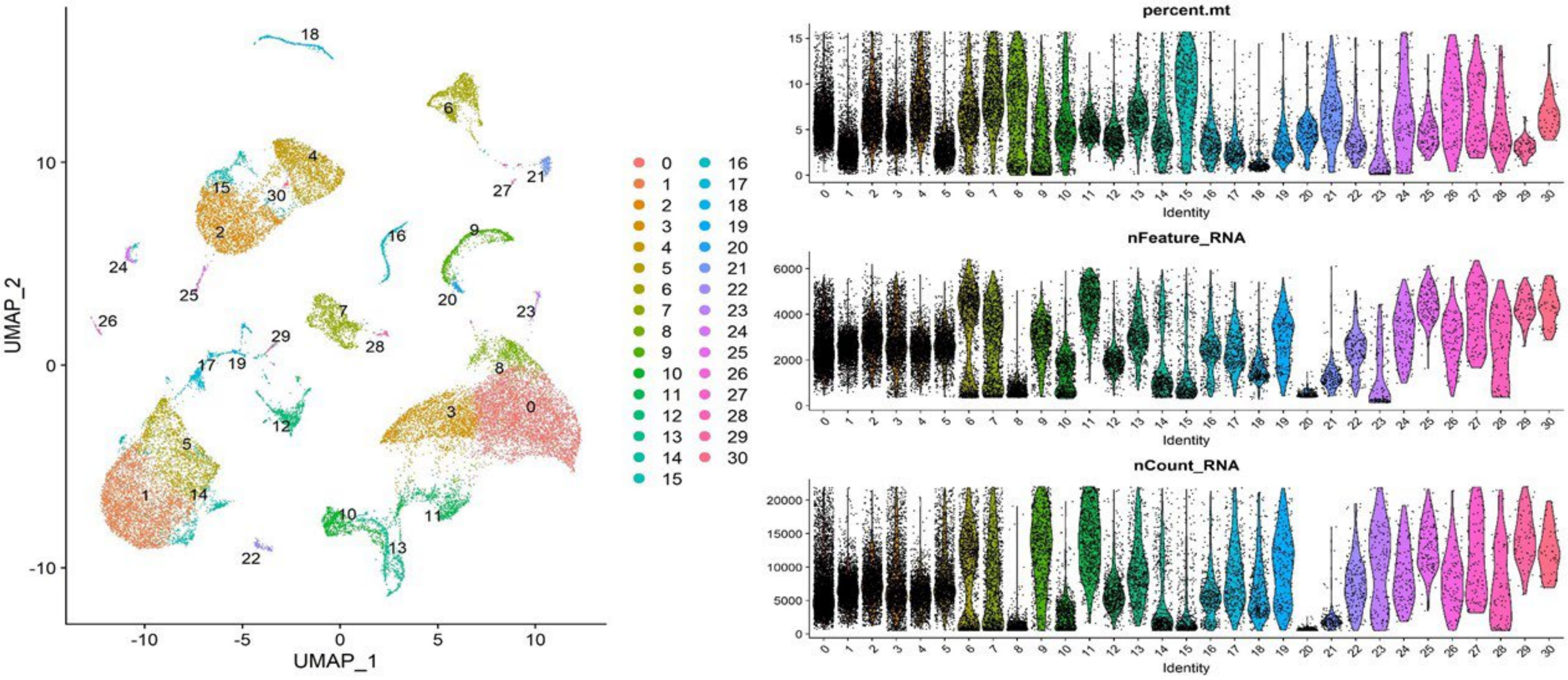

Supplementary Figure S6. Top differentially expressed genes in each microglia cluster, compared to the other microglia cluster (cluster discriminant). Fold changes are reported on the left (bar graph), and percentage of cells expressing each markers on the right (heatmap).

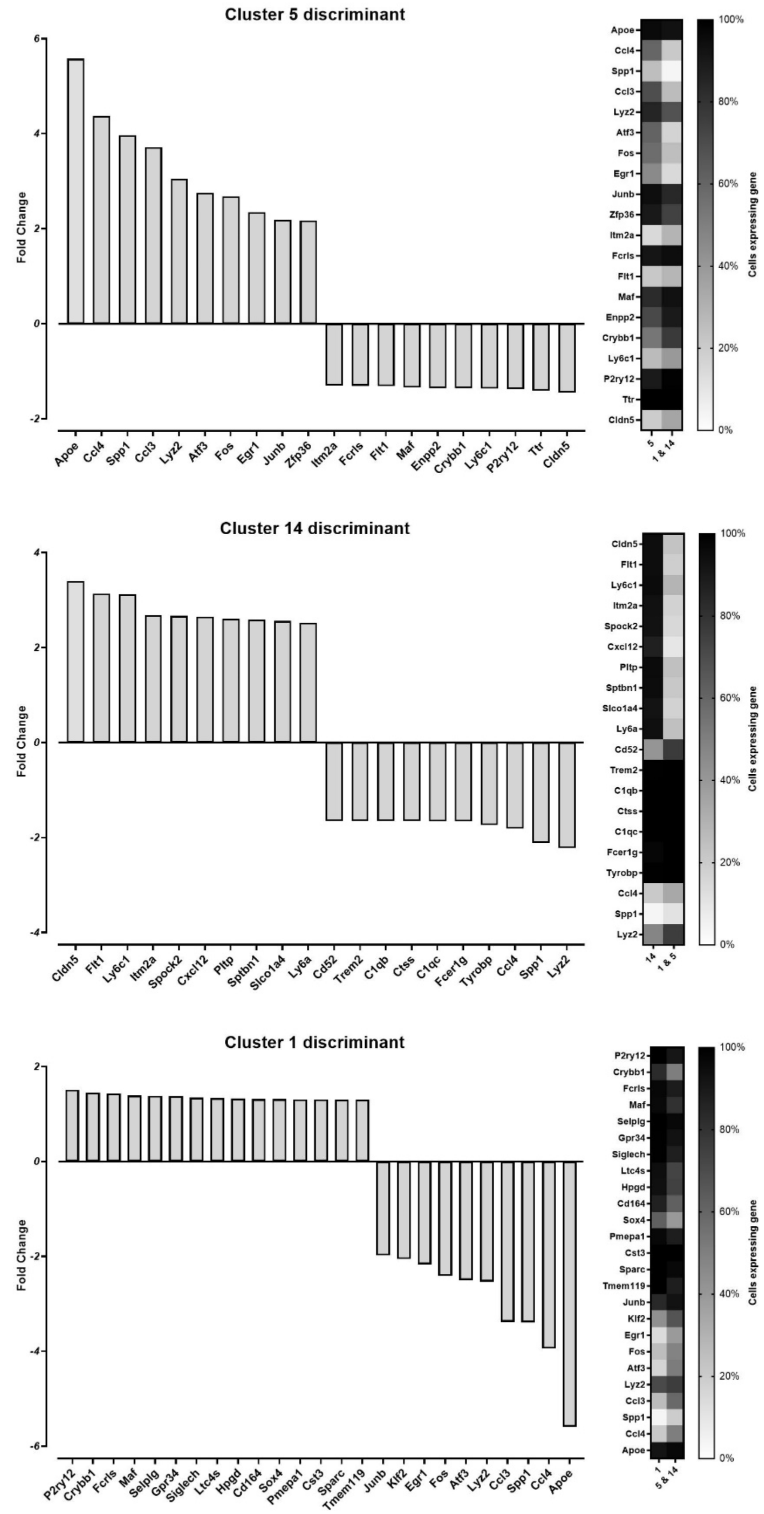

Supplementary Figure S7. Body weight of adult and aged male (M) and female (F) mice fed 0, 2.5, or 5% inulin during the eight-week study.

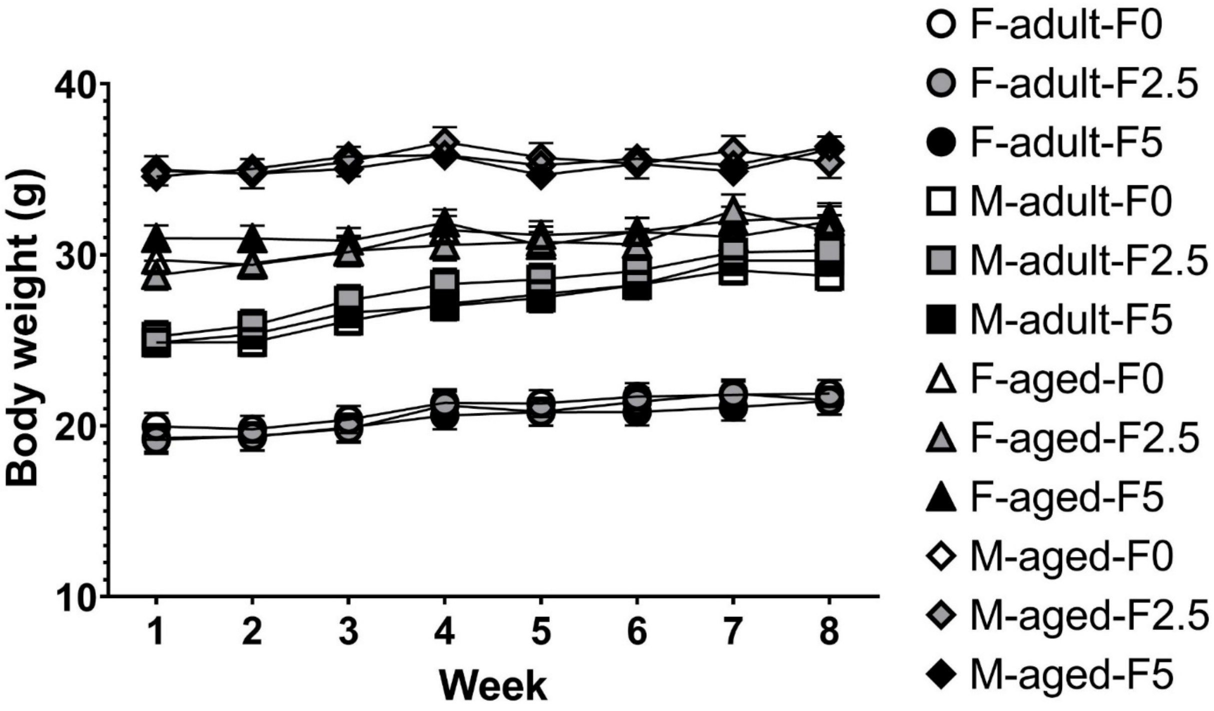

Supplement: Supplementary file 1 [file Data_Sheet_1.pdf]
